# Humeral congenital constriction band in an extremely preterm infant: single-stage circumferential release and neurovascular decompression using multiple Z-plasties

**DOI:** 10.1016/j.jpra.2026.08.019

**Published:** 2026-08-20

**Authors:** Ahmed AlBahlal, Lama A. Alkhwildi, Yara K. Alwathnani, Abdulrahman AlMohaisen

**Affiliations:** a Plastic Surgery Department, Prince Sultan Military Medical City, Riyadh, Saudi Arabia; b Plastic Surgery Department, King Saud Medical City, Riyadh, Saudi Arabia; c College of Medicine, Imam Mohammad Ibn Saud Islamic University (IMISU), Riyadh, Saudi Arabia

**Keywords:** Amniotic band syndrome, Constriction ring syndrome, Humerus, Z-plasty, Preterm infant, Congenital constriction band, Upper extremity, Peripheral nerve

## Abstract

**Background:**

Amniotic band syndrome (ABS) is a rare sporadic disorder in which fibrous bands of amniotic origin encircle and progressively constrict developing fetal structures. Humeral involvement is exceptionally rare and threatens the brachial neurovascular bundle.

**Case presentation:**

A male infant born at 28 weeks and 4 days of gestation was referred at 6 months of chronological age with a single circumferential constriction band of the proximal third of the left humerus (Patterson type 2). Active motion was globally restricted at the shoulder, elbow and wrist, with moderate distal lymphoedema, active finger flexion reduced but present, preserved finger extension and no wrist drop. Perfusion and withdrawal to tactile stimulation were preserved in all digits; the thenar eminence was hypotrophic and the affected hand was smaller than the contralateral hand. Preoperative radiographs confirmed maintained osseous alignment. Surgery was undertaken at 9 months of chronological age, approximately 6.5 months corrected. The band was excised in its entirety using multiple interdigitating Z-plasties; the brachial artery and the radial, median and ulnar nerves were individually identified and decompressed, were normal in calibre, and neurolysis was not required. At 16 months the wounds were healed, all flaps viable, the distal lymphoedema resolved and the groove fully effaced. Electrodiagnostic studies were not performed preoperatively; nerve conduction studies have since been requested.

**Conclusions:**

Single-stage circumferential Z-plasty release with decompression of the brachial neurovascular bundle can be performed safely at the humeral level in an extremely preterm infant. We make no claim regarding neurological recovery, since electrodiagnostic studies were not obtained; the coexisting thenar hypotrophy and reduced hand size raise the possibility of a distal developmental component that decompression would not address, underlining the value of an objective neurological baseline at first presentation.

## Introduction

**Fig. 1:**
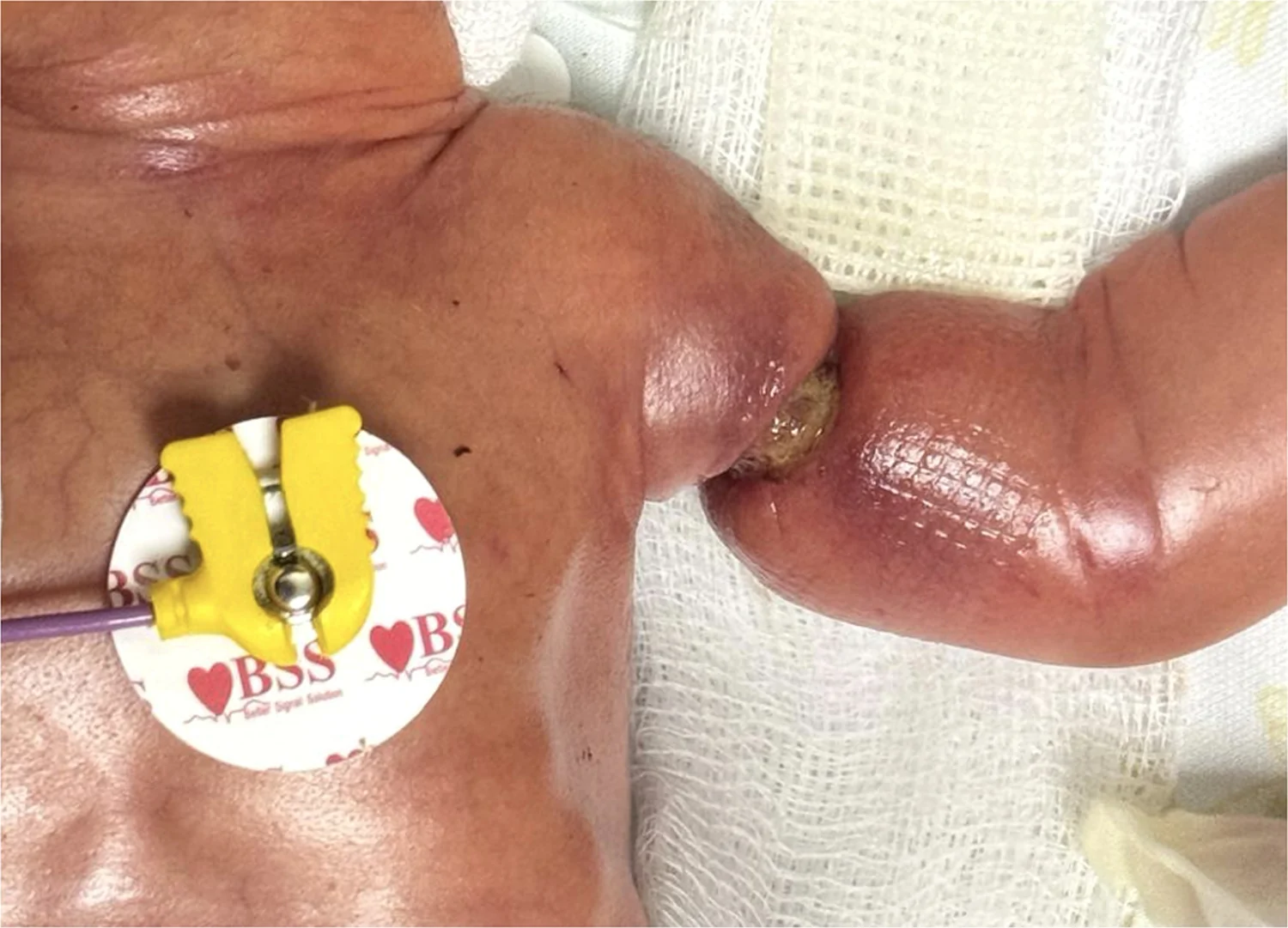
Clinical photograph at initial consultation (age 6 months) demonstrating the circumferential constriction band of the left humerus with associated distal lymphedema of the hand.

Amniotic band syndrome (ABS), also termed constriction ring syndrome or ADAM complex, is a rare, sporadic congenital disorder in which fibrous bands of amniotic origin wrap around and progressively constrict developing fetal structures. It occurs in approximately 1 in 1200 to 1 in 15,000 live births and is associated with preterm delivery in 35–50% of cases.^1^, ^2^, ^3^ The currently favoured exogenous theory proposes that early amnion rupture allows fetal parts to herniate into the chorionic cavity, where they are entrapped and strangulated by fibrous mesodermal strands, while the endogenous theory attributes both the bands and the associated malformations to a single early focal germinal disc disruption.^1^^,^^2^^,^^4^ Constriction bands cluster overwhelmingly at the digital and distal level; humeral involvement accounts for fewer than 5% of extremity cases and carries particular clinical weight given the vulnerability of the brachial neurovascular bundle at this level.^4^^,^^5^ Definitive treatment requires complete excision of the fibrotic band with circumferential Z-plasty reconstruction, and current evidence supports single-stage release.^6^^,^^7^ The series that establish its safety are, however, drawn predominantly from digital and distal rings and give limited guidance at the humeral level, where the neurovascular structures lie immediately deep to the band.^7^^,^^8^ We report a single circumferential constriction band of the proximal third of the left humerus in an infant born at 28 weeks and 4 days of gestation, managed by single-stage multiple Z-plasties with decompression of the brachial neurovascular bundle at 9 months of chronological age, with 16 months of follow-up Fig. 1.

## CASE presentation

A 6-month-old male infant, born prematurely at 28 weeks and 4 days of gestation, was referred to the Plastic and Reconstructive Surgery service for evaluation of a constriction band of the left arm. His corrected age at referral was approximately 3.5 months. His neonatal course was notable for respiratory distress syndrome (RDS) requiring two doses of surfactant, bilateral grade 2 intraventricular hemorrhage (IVH), and a moderate atrial septal defect (ASD) managed conservatively. His weight at the time of referral was 6.1 kg; the mother reported no history of gestational diabetes, teratogenic medication use, or herbal supplement intake during pregnancy.

Clinical examination identified a single circumferential constriction band encircling the left humerus, forming a well-defined groove with the characteristic soft-tissue displacement and moderate distal lymphedema of the hand. The band lay at the proximal third of the humerus, approximately 6 cm proximal to the lateral epicondyle, and corresponded to a Patterson type 2 constriction.^9^

### Preoperative assessment of the affected limb

**Fig. 2:**
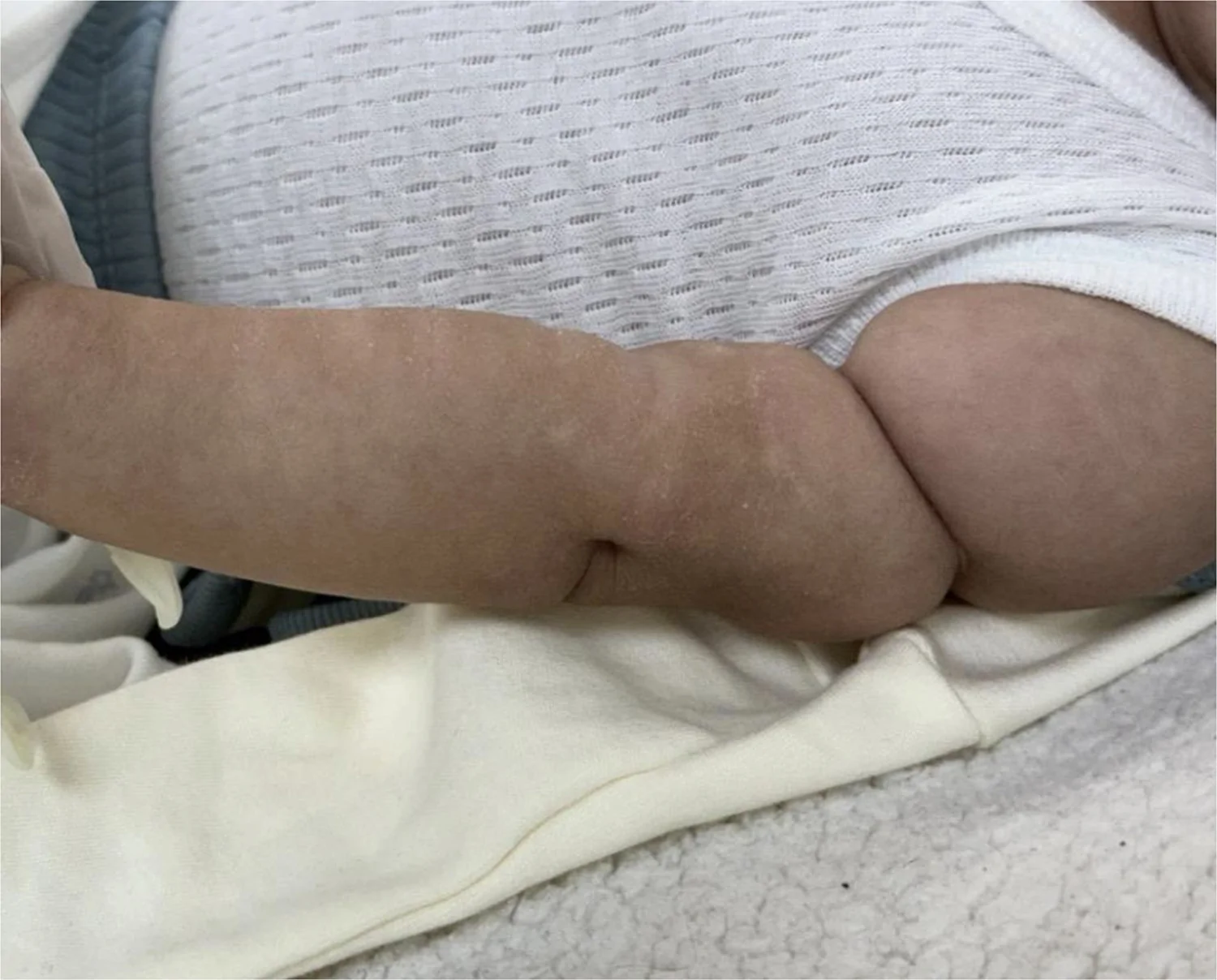
Preoperative view at 9 months of age showing the well-demarcated constriction groove encircling the left humerus with the characteristic soft-tissue displacement, confirming the planned level of circumferential release.

Assessment was performed at referral and repeated on the day of surgery; the findings are set out beside the follow-up findings in Table 1. Active range of motion was globally restricted at the shoulder, elbow and wrist. Active finger flexion was present but reduced in amplitude compared with the contralateral side, whereas active finger extension at the metacarpophalangeal and proximal interphalangeal joints was preserved; there was no wrist drop and no fixed finger flexion deformity. The thenar eminence was hypotrophic relative to the contralateral side and the affected left hand was smaller than the right; the hypothenar eminence was not separately documented. The radial pulse was palpable, capillary refill was under two seconds in all digits, and the infant withdrew appropriately to tactile stimulation of all digits without appreciable asymmetry between the radial, median and ulnar territories. Medical Research Council grading and goniometry are not reliably obtainable at this age, so motor and sensory function were recorded descriptively by observation of spontaneous and stimulus-evoked movement. Nerve conduction studies and electromyography were not performed before surgery; they have since been requested and the results were not available at the time of writing. His mother noted that spontaneous arm movement had been slowly improving since birth, which we record as a subjective parental observation Fig. 2.

**Table 1:** Structured preoperative and follow-up clinical assessment of the affected left upper limb.

Domain	Preoperative (9 months chronological)	Latest follow-up (16 months postoperative)
Constriction band	Single circumferential groove, proximal third of the left humerus; Patterson type 2	Fully effaced; no residual groove
Distal lymphoedema of the hand	Moderate; non-progressive on serial review	Completely resolved
Skin and wound	Intact; no ulceration or maceration	Healed; all Z-plasty flaps viable, no dehiscence or tip necrosis
Perfusion	Radial pulse palpable; capillary refill under 2 s in all digits	Limb well perfused; all flaps viable
Sensation (withdrawal to tactile stimulation)	Withdrawal present in all digits; no appreciable asymmetry between territories	Not separately re-documented
Active shoulder motion	Globally restricted	Not formally re-assessed; mother reports improved spontaneous movement
Active elbow motion	Globally restricted	Not formally re-assessed; mother reports improved spontaneous movement
Active wrist motion	Restricted; no wrist drop	Not formally re-assessed
Active finger flexion	Present but reduced in amplitude compared with the contralateral side	Not formally re-assessed; mother reports improved spontaneous use
Active finger extension (MCP and PIP)	Preserved	Not separately re-documented
Thenar eminence	Hypotrophic relative to the contralateral side	Not formally re-measured
Hypothenar eminence	Not separately documented	Not separately documented
Hand size relative to contralateral	Affected left hand smaller than the right	Not formally re-measured
Fixed finger flexion deformity	Absent	Not separately re-documented
Spontaneous use of the limb	Mother reports slow improvement since birth (subjective)	Mother reports noticeable improvement (subjective)
Nerve conduction studies / EMG	Not performed	Requested; results not available at the time of writing

Assessment in a pre-verbal infant is necessarily observational; Medical Research Council grading and goniometry were not obtainable at this age. MCP, metacarpophalangeal; PIP, proximal interphalangeal; EMG, electromyography.

Preoperative radiographs showed maintained osseous alignment with no evidence of periosteal involvement, cortical erosion or underlying osseous constriction. Cross-sectional imaging of the neurovascular bundle was not obtained, and the decision to operate was made on clinical grounds. Routine blood work, coagulation screen, and cardiology clearance were all satisfactory.

### Rationale for the timing of surgery

**Fig. 3:**
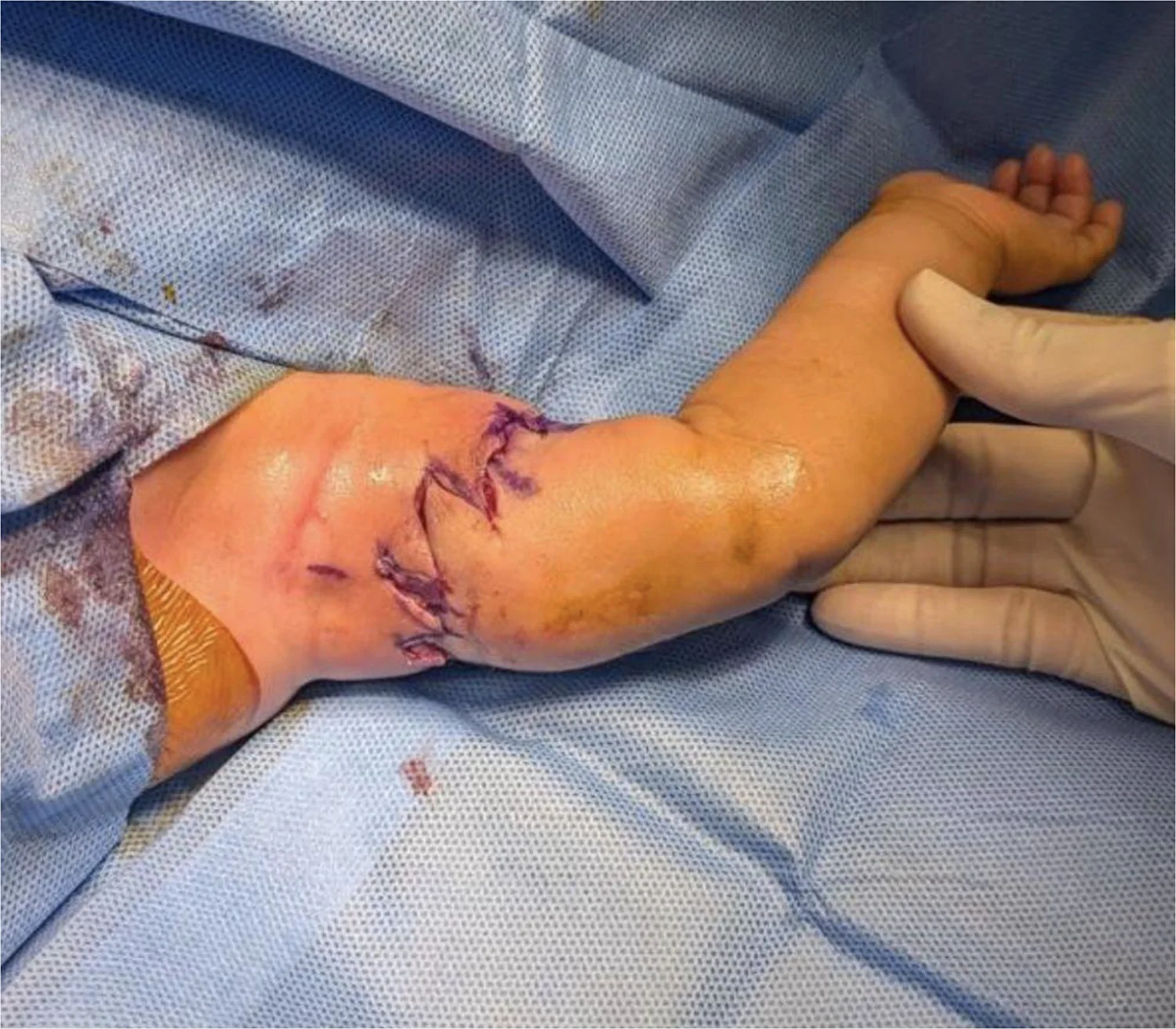
Immediate postoperative photograph following single-stage circumferential release with multiple interdigitating Z-plasties, demonstrating complete band excision and transposition of all flaps with no residual constriction.

The infant was referred at 6 months of chronological age and underwent surgery at 9 months, approximately 6.5 months of corrected age. Two considerations governed this interval. First, there was no indication for emergency release: perfusion was preserved throughout, the lymphoedema was moderate and non-progressive on serial review, there was no skin breakdown, and the limb was managed in the interim with fusidic acid (Fucidin) tulle gras dressings. Second, operation was deferred until satisfactory weight gain had been achieved and the anaesthetic risk conferred by former extreme prematurity, chronic respiratory morbidity and the moderate ASD was judged acceptable. We acknowledge that this interval is longer than in the reported cases in which motor recovery followed decompression, and address the implications below Fig. 3.

## Surgical management

**Fig. 4:**
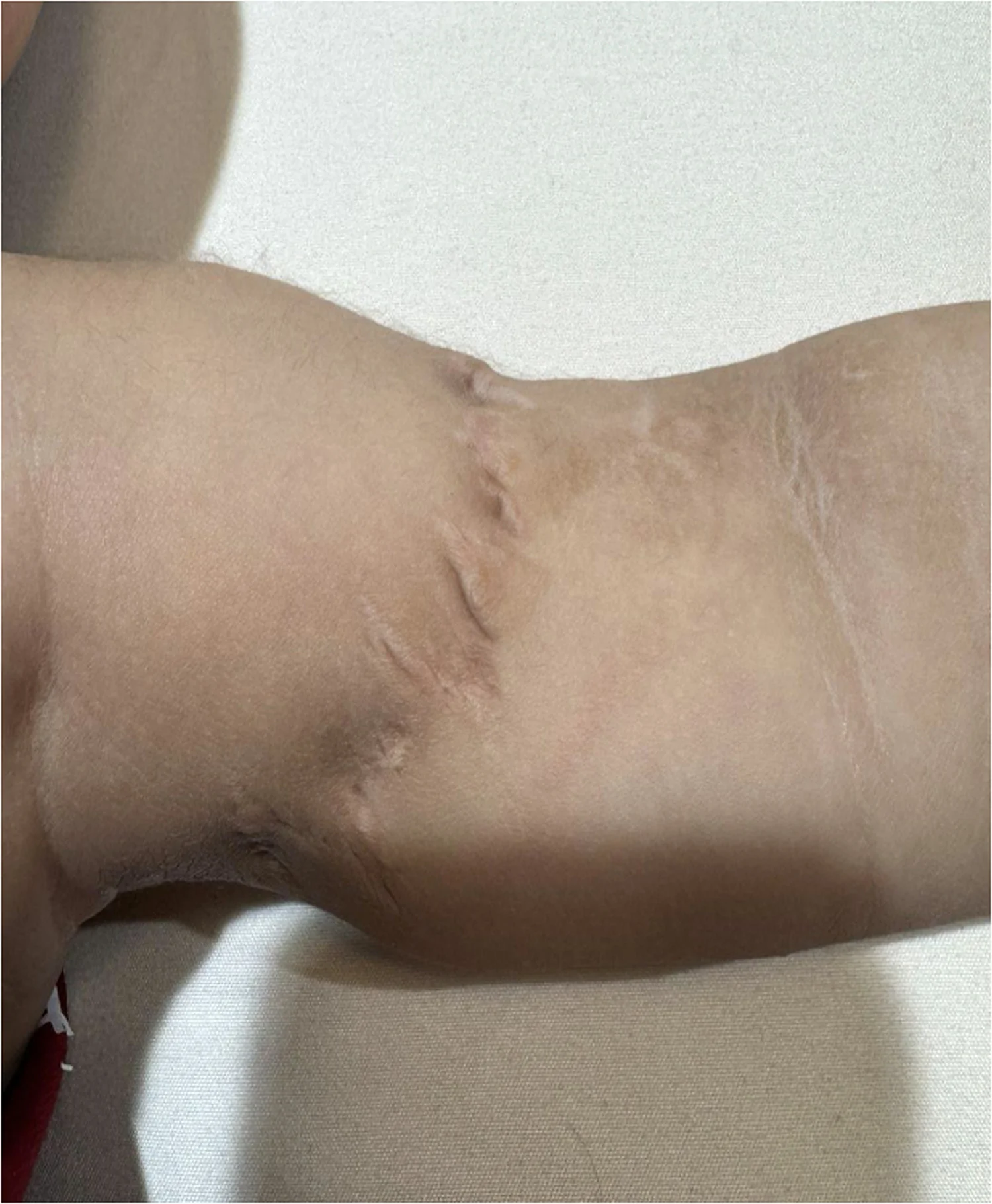
Follow-up photograph at 8 months postoperatively showing well-healed, mature scars with complete effacement of the constriction groove and full resolution of distal lymphedema.

On the day of surgery the patient was placed in the supine position under general anesthesia. Anaesthetic planning took account of his former prematurity, chronic respiratory morbidity and the moderate ASD, and postoperative observation in the paediatric intensive care unit was arranged in advance. The left upper extremity was prepped and draped in the standard sterile manner. Multiple interdigitating Z-plasties with 60-degree flap angles were designed and marked circumferentially around the humeral band. Following tourniquet inflation, a circumferential incision was made through skin and subcutaneous tissue along the marked Z-plasty lines. The skin overlying the groove was de-epithelialised and the entire fibrotic ring was excised to the level of the investing fascia. Careful subcutaneous undermining was then performed to decompress the underlying neurovascular bundle; the brachial artery, cephalic and basilic veins, and the radial, median, and ulnar nerves were each individually identified, preserved, and confirmed intact. All three nerves were normal in calibre and appearance at the level of the constriction, without flattening, indentation or adherence to the overlying fibrotic tissue, and neurolysis was therefore not required. The tourniquet was deflated and capillary refill of under two seconds was confirmed in all digits before closure. Z-plasty flaps were transposed and the wounds were closed with Vicryl Rapid 5–0 and Monocryl 5–0. Estimated blood loss was 5 mL and no transfusion was required. The patient was transferred to the PICU in stable condition Fig. 4.

## Postoperative course and outcomes

Recovery was smooth and uncomplicated. All flaps were well perfused on the first postoperative day, with no dehiscence or tip necrosis, and the patient was discharged home on postoperative day two. He has been followed for 16 months and is now 25 months of age. At latest review the wounds were fully healed, all flaps remained viable, the circumferential groove had been completely effaced and the distal lymphoedema had fully resolved (Table 1). His mother described a noticeable improvement in spontaneous use of the arm; this is a subjective parental observation and is not presented as an objective measure of neurological recovery. Range of motion, thenar bulk and hand size were not formally re-measured, and postoperative nerve conduction studies were requested but not available at the time of writing. He remains under review for physiotherapy and scar management.

## Discussion

Humeral amniotic bands represent one of the rarest manifestations of ABS. In the 70-year institutional series by Foulkes and Reinker, nearly all upper limb lesions were concentrated at the digital level.^5^ and Goldfarb et al. document humeral involvement in fewer than 5% of extremity cases.^4^ What is known of this subset comes almost entirely from isolated reports — Weeks.^10^ Weinzweig and Barr.^11^ and Mohan et al.^12^ — together with the series of Uchida and Sugioka.^13^ and Jones et al.^14^ and the case of Rapp et al.^15^ which are summarised in Table 2.

**Table 2:** Reported arm- and humeral-level congenital constriction bands with neurological involvement.

Study (year)	Age at surgery	Level	Neurological involvement	Technique	Outcome
Weeks (1982).^10^	Infancy; not specified in the indexed record	Arm	Radial, median and ulnar dysfunction	Band excision with decompression of the involved nerves	First description of combined radial, median and ulnar dysfunction from an arm-level band
Uchida & Sugioka (1991).^13^	22 months to 5 years	Proximal to the wrist (4 of 21 patients; 3 with palsy)	Severe median and ulnar motor and sensory dysfunction	Band release with nerve decompression	No meaningful recovery at 9 to 20 years; severe fibrosis on biopsy; nerves flattened and adherent
Weinzweig & Barr (1994).^11^	Infancy; not specified in the indexed record	Arm	Radial, ulnar and median palsies	Single-stage circumferential excision, nerve decompression, multiple large Z-plasties	Significant improvement in neurological function
Jones et al. (2001).^14^	3 months (n = 2); 6 months (n = 1)	Upper extremity	Complete ulnar palsy, clinical and electrophysiological	Band release with nerve decompression	Ulnar palsies persisted at a mean of 7 years; authors advise considering repair or grafting
Rapp et al. (2014).^15^	3 months (release elsewhere); re-exploration at 7 months	Mid-humeral	Flail, non-sensate limb distal to the band	Z-plasty release, then sural nerve grafting of median and ulnar nerves	Nerves atrophic but in continuity; brachial artery disrupted above the elbow; limited recovery
Mohan et al. (2022).^12^	7 days	Proximal to the elbow	Ulnar motor dysfunction	Band release with ulnar, median and radial nerve decompression	Early evidence of ulnar motor function at 5.5 months
Present case	9 months chronological; approximately 6.5 months corrected	Proximal third of the left humerus	Reduced amplitude of active finger flexion with preserved sensation and perfusion; thenar hypotrophy and reduced hand size; no complete palsy	Single-stage circumferential release, multiple interdigitating 60-degree Z-plasties, decompression of the brachial artery and the radial, median and ulnar nerves	Complete excision; lymphoedema resolved; contour restored at 16 months; neurological outcome not determined, electrodiagnostic studies pending

Ages are chronological unless otherwise stated. The 1982 and 1994 reports predate structured abstracts and the exact age at operation is not given in the indexed record.

### What this report adds

Single-stage circumferential release is not novel and we claim no new operation. Habenicht et al. reported a ten-year experience of one-step correction by complete circular resection with linear circumferential closure.^7^ and Dufournier et al. 14 patients of mean age 13.3 months treated by one-stage resection with direct circular suture, with a mean follow-up of 3.9 years and no vascular, neurological or scar-related complications.^8^ The distinctions we draw are of anatomical level, closure design and patient population. Both series address predominantly distal and digital rings, where the structures at risk beneath the band are digital neurovascular bundles and the procedure is essentially a soft-tissue one; at the humeral level the brachial artery and the three major nerves lie immediately deep to the constriction, so excision to the investing fascia cannot be undertaken without deliberate identification and decompression of each, which converts a contour correction into a neurovascular procedure. Both series also dispense with Z-plasty in favour of a direct circular suture line, whereas we retained interdigitating Z-plasties on the reasoning that a linear circumferential scar overlying a growing segment containing the neurovascular bundle carries a theoretical risk of secondary constriction with growth — a rationale rather than a demonstrated advantage. Neither series includes an extremely preterm infant, and much of what we report concerns how the timing and conduct of the operation were shaped by his comorbidities.

The co-occurrence of ASD, bilateral IVH, and extreme prematurity in our patient fits within the 35–50% prematurity rate documented across ABS series and lends credence to the possibility of an underlying intrinsic embryopathic process, a distinction that bears on family counseling though it does not alter the operative approach.^3^^,^^4^

### Neurological considerations and the timing of decompression

The published experience points to a time-dependent window for recovery. Uchida and Sugioka found no meaningful motor or sensory recovery at 9 to 20 years in patients decompressed between 22 months and 5 years of age, with severe fibrosis on muscle biopsy and nerves that were flattened and adherent.^13^ Jones et al. reported persistent ulnar palsy at a mean of 7 years despite release at 3 to 6 months, and advised considering resection with repair or grafting where exploration reveals significant compression.^14^ and Rapp et al. described a mid-humeral case requiring sural nerve grafting at 7 months.^15^ Against these, Weinzweig and Barr reported significant improvement in neurological function after single-stage correction in an infant.^11^ and Mohan et al. early ulnar motor function following decompression at 7 days of age.^12^

Our patient does not permit a contribution to this question. He did not present with a complete palsy of any of the three nerves: withdrawal to tactile stimulation was present in all digits without appreciable asymmetry, perfusion was intact, finger extension was preserved, there was no wrist drop, and finger flexion was reduced rather than absent. The restricted range of motion is as plausibly attributable to the mechanical effect of the band and the associated lymphoedema as to nerve dysfunction, and we did not obtain the studies that would have distinguished them; the intraoperative findings are consistent with this, all three nerves being normal in calibre without the flattening and adherence described by Uchida and Sugioka.^13^ We can state that the band was excised completely, that the neurovascular structures were decompressed and macroscopically normal, and that the lymphoedema resolved and limb contour was restored and maintained at 16 months. We cannot state, and no longer state, that nerve function was compromised preoperatively or improved as a consequence of decompression. Prospectively we would endorse the algorithm of Weinzweig and Barr: complete neurological examination even in the infant, electrodiagnostic studies wherever nerve dysfunction is suspected, early single-stage excision, and early decompression of all major nerve trunks where dysfunction is present.^11^

### Thenar hypotrophy and a possible coexisting distal developmental deficit

Constriction ring syndrome may coexist with congenital hypoplasia of the distal upper-extremity nerves rather than being purely compressive, suggested clinically by a smaller affected hand, hypotrophy and flattening of the thenar and hypothenar eminences, and finger flexion deformities from intrinsic muscle dysfunction. Two of these features were present in our patient, and two interpretations cannot be separated on our data: chronic median nerve compression at the level of the band producing denervation atrophy of the thenar musculature, or a coexisting developmental deficit of the distal limb. Several features favour the latter, or at least a contribution from it: the generalised reduction in hand size is not readily explained by a lesion confined to the humeral level; withdrawal to tactile stimulation was symmetrical, which would be unusual in a median lesion severe enough to produce visible thenar wasting; and the nerves were normal in calibre at exploration. If a developmental component is present, thenar bulk and hand size would not be expected to normalise after release, and improvement in use of the limb cannot be attributed to decompression.^13^^,^^14^ This makes electrodiagnostic assessment more rather than less important, and we recommend that bilateral comparative assessment of the hand form part of the preoperative evaluation of any proximal constriction band.

### Technical considerations

Upton and Tan reported 95% excellent or good outcomes in 58 patients treated with Z-plasty combined with subcutaneous fat advancement.^6^ while both Habenicht et al.^7^ and Prasetyono and Sitorus.^16^ have confirmed that single-stage circumferential release can be performed safely and without recurrence, owing to preservation of the deep musculocutaneous perforators that sustain distal perfusion. Dufournier et al. reached the same conclusion using direct circular closure.^8^ In our patient the flaps healed without tip necrosis and the contour was maintained at 16 months without recurrent constriction.

## Limitations

Electrodiagnostic studies were not performed before surgery and those requested subsequently were unavailable at the time of writing, so we can neither confirm that the observed weakness was neurogenic nor distinguish compression at the level of the band from a distal developmental deficit. Motor and sensory function were assessed descriptively rather than by Medical Research Council grading or goniometry, which are not reliably obtainable in a pre-verbal infant, and range of motion, thenar bulk and hand size were not formally re-measured at follow-up; the hypothenar eminence was not documented at presentation. No cross-sectional imaging was obtained, and dedicated bilateral comparative photographs of the hands and wrists were not taken and could not subsequently be obtained because the family resides in another city. Part of the reported improvement derives from parental observation and is therefore subjective. Finally, although 16 months exceeds the follow-up in most reports of this lesion, it is short relative to the timescale over which neurological outcome, scar quality and secondary constriction with growth declare themselves.^13^^,^^14^ and no inference regarding Z-plasty against direct circular closure can be drawn from a single case.

## Conclusion

Congenital constriction band at the humeral level is exceptionally rare, and the published experience consists almost entirely of isolated case reports. This case demonstrates that single-stage circumferential release with multiple interdigitating Z-plasties and decompression of the brachial neurovascular bundle can be performed safely in an extremely preterm infant with significant cardiorespiratory and neurological comorbidity, achieving complete excision of the band, resolution of distal lymphoedema and restoration of limb contour, maintained at 16 months. Whether the procedure altered neurological function cannot be determined, since baseline electrodiagnostic studies were not obtained, and the coexisting thenar hypotrophy and reduced hand size raise the possibility of a distal developmental deficit that release at the level of the band would not correct. The principal lesson is the value of establishing an objective neurological baseline, clinical and electrodiagnostic, at first presentation, so that the effect of release can afterwards be assessed rather than assumed.

## Ethical approval

Ethical approval was not required for this case report.

## Patient consent statement

Written informed consent for publication of clinical information and operative photographs was obtained from the patient's legal guardian (father).

## Declaration of competing interest

The authors declare that they have no conflicts of interest.
